# Estimation of Information Flow-Based Causality with Coarsely Sampled Time Series

**DOI:** 10.3390/e28010034

**Published:** 2025-12-26

**Authors:** X. San Liang

**Affiliations:** 1 Department of Atmospheric and Oceanic Sciences, Fudan University, Shanghai 200438, China; xsliang@fudan.edu.cn; 2 The Artificial Intelligence Department, Division of Frontier Research, Southern Marine Science and Engineering Guangdong Laboratory (Zhuhai), Zhuhai 519082, China

**Keywords:** quantitative causality, information flow, coarsely sampled time series, synchronization, Rössler system, Lie group, Frobenius-Perron operator

## Abstract

The past decade has seen growing applications of the information flow-based causality analysis, particularly with the concise formula of its maximum likelihood estimator. At present, the algorithm for its estimation is based on differential dynamical systems, which, however, may raise an issue for coarsely sampled time series. Here, we show that, for linear systems, this is suitable at least qualitatively, but, for highly nonlinear systems, the bias increases significantly as the sampling frequency is reduced. This study provides a partial solution to this problem, showing how causality analysis can be made faithful with coarsely sampled series, provided that the statistics are sufficient. The key point here is that, instead of working with a Lie algebra, we turn to work with its corresponding Lie group. An explicit and concise formula is obtained, with only sample covariances involved. It is successfully applied to a system comprising a pair of coupled Rössler oscillators. Particularly remarkable is the success when the two oscillators are nearly synchronized. As more often than not observations may be scarce, this solution, albeit partial, is very timely.

## 1. Introduction

Causality analysis is a fundamental task in scientific research. Though traditionally formulated as a statistical problem (see, for example, the classics by Granger [1], Pearl [2], and Rubin [3,4]) in data science and computer science, among other disciplines, formalisms within the framework of dynamical systems have also been established; refer to a focus issue of *Chaos* [5] for relevant references. Particularly, in terms of information flow/transfer, it has been argued that causality is “a real notion in physics that can be derived *ab initio*” [6]. A comprehensive study with generic systems has been conducted, and explicit formulas have been attained in closed form [6,7] with the aid of the Frobenius–Perron operator (e.g., [8]), a technique also exploited in similar studies (e.g., [9]). These formulas have been validated with benchmark systems such as the baker transformation, Hénon map, etc., and have been applied successfully to real-world problems in diverse disciplines, such as global climate change, dynamic meteorology, land–atmosphere interaction, data-driven prediction, near-wall turbulence, neuroscience, financial analysis, and quantum information; see [10] for a brief review of the applications and  [11,12,13,14,15,16] for some updates.

For the purpose of this study, we first give a brief introduction to the theory within the framework of a differential dynamical system (also available for discrete-time mappings; see [6]). Let

(1) $$\begin{array}{c} \frac{dx}{dt}=F\left(x,t\right)+B\left(x,t\right)\dot{w}, \end{array}$$

be a *d*-dimensional continuous-time stochastic system for $x=(x_{1},\ldots ,x_{d})$ (we do not distinguish notations for random and deterministic variables), where $F=(F_{1},\ldots ,F_{d})$ may be arbitrary nonlinear differentiable functions of x and *t*, $\dot{w}$ is a vector of white noise, and $B=(b_{ij})$ is the matrix of perturbation amplitudes, which may also be any differentiable functions of x and *t*. This setting, particularly the setting in the absence of stochasticity, avails us of the arsenal from physics in approaching the problem. For example, the concept of symmetry has been found to play an important role [17]. Using the Frobenius–Perron operator, Liang (2016) [6] proved that the rate of information flowing from $x_{j}$ to $x_{i}$ (in nats per unit time) is

(2) $$\begin{array}{ccc} T_{j\to i} & = & -E\left[\frac{1}{\rho_{i}}\int_{R^{d-2}}\frac{\partial (F_{i}\rho_{\setminus \;j})}{\partial x_{i}}dx_{\setminus \;i\setminus \;j}\right]+\frac{1}{2}E\left[\frac{1}{\rho_{i}}\int_{R^{d-2}}\frac{\partial^{2}\left(g_{ii}\rho_{\setminus \;j}\right)}{\partial x_{i}^{2}}dx_{\setminus \;i\setminus \;j}\right],\\ & = & -\int_{R^{d}}\rho_{j|i}\left(x_{j}|x_{i}\right)\frac{\partial (F_{i}\rho_{\setminus \;j})}{\partial x_{i}}dx+\frac{1}{2}\int_{R^{d}}\rho_{j|i}\left(x_{j}|x_{i}\right)\frac{\partial^{2}\left(g_{ii}\rho_{\setminus \;j}\right)}{\partial x_{i}^{2}}dx, \end{array}$$

where $dx_{\setminus \;i\setminus \;j}$ signifies $dx_{1}\ldots dx_{i-1}dx_{i+1}\ldots dx_{j-1}dx_{j+1}\ldots dx_{n}$, *E* stands for mathematical expectation, $g_{ii}=\sum_{k=1}^{n}b_{ik}b_{ik}$, $\rho_{i}=\rho_{i}\left(x_{i}\right)$ is the marginal probability density function (pdf) of $x_{i}$, $\rho_{j|i}$ is the pdf of $x_{j}$ conditioned on $x_{i}$, and $\rho_{\setminus \;j}=\int_{R}\rho (x)dx_{j}$. The algorithm for the information flow-based causal inference is as follows: if $T_{j\to i}=0$, then $x_{j}$ is not causal to $x_{i}$; otherwise, it is causal, and the absolute value measures the magnitude of the causality from $x_{j}$ to $x_{i}$. This is guaranteed by a property called the “principle of nil causality”. Another property, as proven by Liang (2018) (see [10]), regards the invariance upon coordinate transformation, indicating that the obtained information flow (IF) is an intrinsic property in nature. Also established is that [6], for a linear model, i.e., for F(x,t)=Ax, with $A=(a_{ij})$ and $B=(b_{ij})$ being constant matrices in (1), the information flow rate from $x_{j}$ to $x_{i}$ is

$$\begin{array}{c} T_{j\to i}=a_{ij}\frac{\sigma_{ij}}{\sigma_{ii}}, \end{array}$$

where $\sigma_{ij}$ is the population covariance of $x_{i}$ and $x_{j}$. By this, it follows that, in the linear sense, causation implies correlation, but not vice versa. In an explicit expression, this corollary expresses mathematically the debate on causation vs. correlation ever since George Berkeley (1710) [18].

In the case with only *d* time series $x_{1},x_{2},\ldots ,x_{d}$, the quantitative causality, i.e., the IF, between them can be estimated using maximum likelihood estimation (see [19,20]). Under the assumption of a linear system with additive noise, the maximum likelihood estimator (mle) of (2) for $T_{j\to i}$ is [20]

(3) $$\begin{array}{c} {\hat{T}}_{j\to i}=\frac{1}{\det C}\cdot \sum_{\nu =1}^{d}\Delta_{j\nu }C_{\nu ,di}\cdot \frac{C_{ij}}{C_{ii}}, \end{array}$$

where $C_{ij}$ is the sample covariance between $x_{i}$ and $x_{j}$, $\Delta_{ij}$ are the cofactors of the matrix $C=(C_{ij})$, and $C_{i,dj}$ is the sample covariance between $x_{i}$ and a series derived from $x_{j}$ using the Euler forward differencing scheme: ${\dot{x}}_{j,n}=\left(x_{j,n+k}-x_{j,n}\right)/\left(k\Delta t\right)$, with being k≥1 some integer. Equation (3) is rather concise in form, involving only the common statistics, i.e., sample covariances. The transparent formula makes causality analysis, which otherwise would be complicated, very easy and computationally efficient. Note, however, that Equation (3) cannot replace Equation (2); it is merely the maximum likelihood estimator (mle) of the latter. Statistical significance tests can be performed for the estimators. This is achieved with the aid of a Fisher information matrix. See [19,20] for details.

Originally, the formalism was established within the framework of a differential system; in other words, it is with infinitesimal time increments. (The formalism with discrete mappings was also established by Liang (2016) [6], but no estimation has ever been made so far.) A question naturally arises about its applicability in the case of coarsely sampled time series. Indeed, it is not unusual that the given series may be coarsely sampled due to limited observations. As will be seen in the following section, this may pose a problem for nonlinear systems if the sample interval is large. This paper henceforth attempts to address this issue in the original linear framework. In the following, we first check the applicability of (3) for series from a linear system and a highly nonlinear system (Section 2), with a variety of sampling intervals. A new approach is presented in Section 3, which is then utilized to reconduct the causal inferences in Section 2. Some remaining issues are discussed in Section 5.

## 2. The Issue with Coarsely Sampled Series

### 2.1. Time Series from Linear Systems

**Figure 1:**
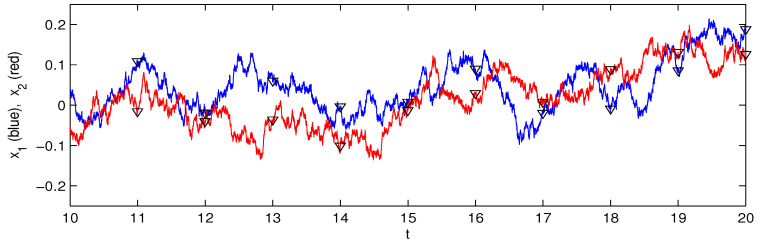
A segment of the sample path generated by Equation (4), with a time step Δt=0.001 (blue: $x_{1}$; red: $x_{2}$). The time spans from 0 to 100. The triangles mark the sampling points. As we are only interested in the limit IF as t→∞, the first 5000 steps, i.e., those for t≤5, are discarded.

We first test the applicability of (3), as the sampling interval increases, with a well-studied linear system whose IF rates have been found half-analytically. This is the validation example in  [19]:

(4a) $$\begin{array}{ccc} \frac{dx_{1}}{dt} & = & -x_{1}+0.5x_{2}+0.1{\dot{w}}_{1} \end{array}$$

(4b) $$\begin{array}{ccc} \frac{dx_{2}}{dt} & = & -x_{2}+0.1{\dot{w}}_{2} \end{array}$$

where ${\dot{w}}_{i}$, i=1,2 are components of independent white noise. It has been shown that the rates of information flow per unit time are $T_{2\to 1}\to 0.11$ as t→∞, and $T_{1\to 2}=0$ for all *t*, reflecting accurately the one-way causality from $x_{2}$ to $x_{1}$. Now, using the same sample path as that in Liang (2014) [19], we resample the series with low frequencies to obtain new series. (Note that, because of the pseudorandom number generator, the generated sample path using the normal differencing scheme may not be satisfactory. To see whether the obtained sample path is correct, one may check the resulting covariances, which can be rather accurately obtained by solving a deterministic ODE. Here, the data for the generated sample path can be downloaded from http://www.ncoads.org/article/show/68.aspx under the item PRE_2014.dat (accessed on 1 January 2020).) Shown in Figure 1 is part of the sample path, with triangles marking the sampling points.

The computed IFs for different sampling intervals are listed in Table 1. We also compute the confidence intervals at a level of 90% (at a significance level of 0.1). First, the estimators ${\hat{T}}_{2\to 1}$ for all SIs here are significantly distinct from zero, while those in the opposite case, ${\hat{T}}_{1\to 2}$, are not significant at a level of 90%. Thus, the causality in a qualitative sense can be faithfully recovered even with very low sampling frequencies (large SIs). (In fact, even with SI = 1000, the result is still correct; we do not consider cases beyond SI = 500 since the sample size is too small for SI > 500, resulting in insufficient statistics.)

Since this example actually has a half-analytical solution ($T_{2\to 1}\approx 0.11$, $T_{1\to 2}=0$), further conclusions can be made about the computed results. Generally, the result of ${\hat{T}}_{1\to 2}$ appears satisfactory. For ${\hat{T}}_{2\to 1}$, it is rather accurate for SI ≤ 100. Beyond 100, it is no longer accurate.

### 2.2. Time Series from Synchronized Chaotic Oscillators

**Figure 2:**
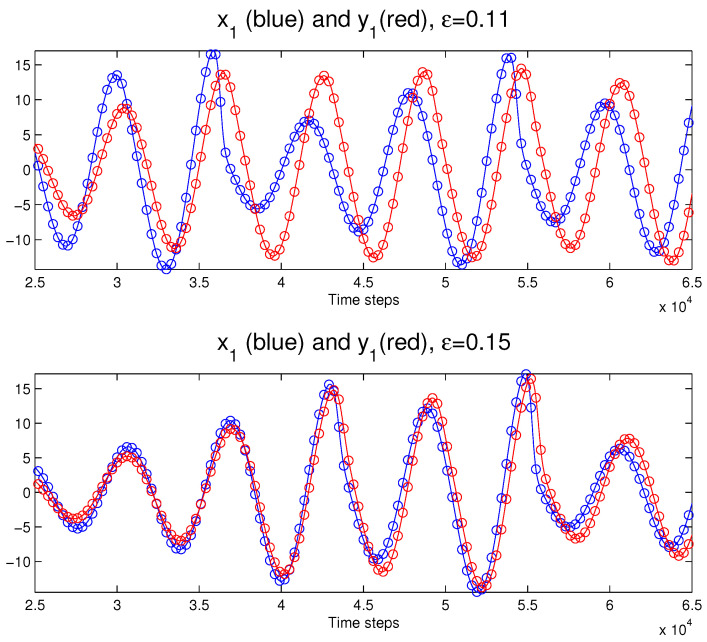
Part of the time series of $x_{1}$ and $y_{1}$ of the coupled Rössler systems for ε=0.11 (**top**) and ε=0.15 (**bottom**). The circles indicate the sampling points (every 300 steps here, corresponding to 18 points in each period). The two oscillators become synchronized as ε≥0.15.

The following example is from the synchronization problem as examined by Paluš et al. [21]. The system is composed of two Rössler oscillators, $x=(x_{1},x_{2},x_{3})$ and $y=(y_{1},y_{2},y_{3})$, where

(5a) $$\begin{array}{ccc} & & \frac{dx_{1}}{dt}=-\omega_{1}x_{2}-x_{3}, \end{array}$$

(5b) $$\begin{array}{ccc} & & \frac{dx_{2}}{dt}=\omega_{1}x_{1}+0.15x_{2}, \end{array}$$

(5c) $$\begin{array}{ccc} & & \frac{dx_{3}}{dt}=0.2+x_{3}\left(x_{1}-10\right), \end{array}$$

is the master system, and

(6a) $$\begin{array}{ccc} & & \frac{dy_{1}}{dt}=-\omega_{2}y_{2}-y_{3}+\varepsilon \left(x_{1}-y_{1}\right), \end{array}$$

(6b) $$\begin{array}{ccc} & & \frac{dy_{2}}{dt}=\omega_{2}y_{1}+0.15y_{2}, \end{array}$$

(6c) $$\begin{array}{ccc} & & \frac{dy_{3}}{dt}=0.2+y_{3}\left(y_{1}-10\right), \end{array}$$

is the driven one. Following [21], we choose $\omega_{1}=1.015$ and $\omega_{2}=0.985$. Using the Runge–Kutta scheme and choosing a time step Δt=0.001, the coupled six-dimensional system can be solved rather accurately with different ε. Figure 2 plots the solutions of $x_{1}$ and $y_{1}$ when the coupling strength ε=0.11 (upper panel) and ε=0.15 (lower panel). As shown in the latter case, the two subsystems become synchronized if ε≥0.15. Again, we choose to study the problem for t∈[0,100] ($10^{5}$ time steps in total).

**Figure 3:**
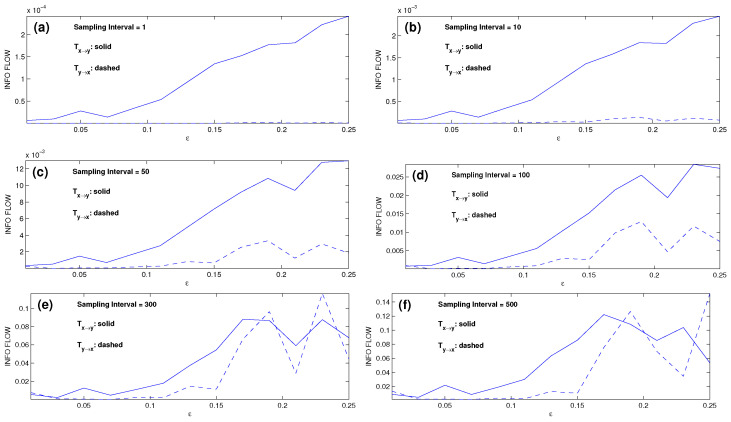
Absolute information flow rates $|{\hat{T}}_{x\to y}|$ and $|{\hat{T}}_{y\to x}|$ (dashed) between the two Rössler oscillators as functions of the coupling coefficient ε with different sampling intervals ((**a**)–(**f**) correspond to SI = 1, 10, 50, 100, 300, 500, respectively). Units are nats per unit time. With the preset causality, the dashed line should coincide with the abscissa.

For each ε, we generate six time series of $10^{5}$ steps and evaluate the IFs according to Equation (3) (k=1 is chosen). The IFs as functions of ε are then obtained, and they are plotted in Figure 3a, which accurately shows that the master is x, and y is the slave. An interesting observation is that this causality inference works even when the two oscillators are nearly synchronized as ε>0.15, demonstrating the power of this rigorously formulated causality analysis.

We subsample the series at every SI step, SI = 10, 50, 100, 300, 500, and reconduct the computation using the same scheme. The resulting IF rates are shown in Figure 3b–f, respectively. With the preset causality, the dashed line should be the zero line. Clearly, the causal inference works well for SI ≤ 10. The computed IF becomes biased for SI ≥ 50, and the bias grows significantly as the SI increases. If we focus on ε≤0.15, i.e., when the systems are not synchronized (see [21]), the causal inference still functions well for SI ≤ 50. If the synchronized cases are taken into account (ε > 0.15), then the inferences in the cases for 50≤SI≤100 are significantly biased, and those for SIs exceeding 300, corresponding to an approximate sampling frequency of 20 per period, are no longer correct.

## 3. Approaching a Partial Solution

As shown above, if the sampling frequency of the time series is low, the resulting linear IF for nonlinear series may be biased. Indeed, in the case with high nonlinearity, the linear assumption is always easy to blame. While theoretically it is not a problem (causality is guaranteed, as it is proven in a theorem), we agree that, before a fully nonlinear algorithm is developed, this will be a continuing issue. What we want to show here is, how much room there is for improvement. At present, the algorithm documented in [19], and later in [20], is based on the Bernstein–Euler differencing scheme, which is, of course, very rudimentary due to the first-order differencing. If a time series is coarsely sampled, the error could be large.

A theorem established by Liang (2008) [7] reads that, if the noise is additive in Equation (1), i.e., if B is a constant matrix, then the noise itself does not appear in the formula of $T_{j\to i}$. Thus, under the additive noise assumption, we can estimate the IF within the framework of a deterministic system. In this case, note that the linear equation set can be solved for an interval [t,t+Δt], regardless of the size of Δt. This gives insight regarding a solution to the low sampling frequency problem.

Consider

(7) $$\begin{array}{c} \frac{dx}{dt}=f+Ax, \end{array}$$

where $A=(a_{ij})$ is a d×d matrix. Let us assume that f=0, since the time series can always be pretreated by removing the linear trend, and it has been proven that this removal does not alter the IF rates. In this case, on the interval [t,t+Δt], we actually have a mapping $\Phi :R^{d}\to R^{d}$ that takes the state x(t) to the state x(t+Δt) at t+Δt, with the propagating operator

(8) $$\begin{array}{c} \Phi =e^{A\Delta t}=e^{\left[\begin{array}{ccc} a_{11} & \ldots & a_{1d}\\ \vdots & \ddots & \vdots \\ a_{d1} & \ldots & a_{dd} \end{array}\right]\Delta t}\equiv \left[\begin{array}{ccc} \alpha_{11} & \ldots & \alpha_{1d}\\ \vdots & \ddots & \vdots \\ \alpha_{d1} & \ldots & \alpha_{dd} \end{array}\right]. \end{array}$$

It is not easy to estimate $a_{ij}$, but it is easy to estimate $\alpha_{ij}$ instead, by observing the relation

(9) $$\begin{array}{ccc} & & \left[\begin{array}{ccc} \alpha_{11} & \ldots & \alpha_{1d}\\ \vdots & \ddots & \vdots \\ \alpha_{d1} & \ldots & \alpha_{dd} \end{array}\right]x\left(n\right)=x\left(n+1\right),\;\;n=0,1,2,...,N. \end{array}$$

This is written in matrix form as

$$\begin{array}{c} \left[\begin{array}{ccc} x_{1}\left(0\right) & \ldots & x_{d}\left(0\right)\\ \vdots & \ddots & \vdots \\ x_{1}\left(N-1\right) & \ldots & x_{d}\left(N-1\right) \end{array}\right]\left[\begin{array}{c} \alpha_{i1}\\ \vdots \\ \alpha_{id} \end{array}\right]=\left[\begin{array}{c} x_{i}\left(1\right)\\ \vdots \\ x_{i}\left(N\right) \end{array}\right] \end{array}$$

for i=1,…,d. Averaging all rows of the algebraic equation set, and subtracting the mean from each row, we get

$$\begin{array}{c} \left[\begin{array}{ccc} x_{1}\left(0\right)-{\bar{x}}_{1} & \ldots & x_{d}\left(0\right)-{\bar{x}}_{d}\\ \vdots & \ddots & \vdots \\ x_{1}\left(N-1\right)-{\bar{x}}_{1} & \ldots & x_{d}\left(N-1\right)-{\bar{x}}_{d} \end{array}\right]\left[\begin{array}{c} \alpha_{i1}\\ \vdots \\ \alpha_{id} \end{array}\right]=\left[\begin{array}{c} x_{i}\left(1\right)-{\bar{x}}_{i+}\\ \vdots \\ x_{i}\left(N\right)-{\bar{x}}_{i+} \end{array}\right], \end{array}$$

where ${\bar{x}}_{i}=\frac{1}{N}\sum_{n=0}^{N-1}x_{i}\left(n\right)$, ${\bar{x}}_{i+}=\frac{1}{N}\sum_{n=1}^{N}x_{i}\left(n\right)$, i.e., the series $\left\{x_{i+}\left(n\right)\right\}$ is the series $\left\{x_{i}\left(n\right)\right\}$ advanced by one step. Let *i* run through {1,2,…,d}. We have the following *d* overdetermined equation sets:

(10) $$\begin{array}{c} \left[\begin{array}{ccc} x_{1}\left(0\right)-{\bar{x}}_{1} & \ldots & x_{d}\left(0\right)-{\bar{x}}_{d}\\ \vdots & \ddots & \vdots \\ x_{1}\left(N-1\right)-{\bar{x}}_{1} & \ldots & x_{d}\left(N-1\right)-{\bar{x}}_{d} \end{array}\right]\left[\begin{array}{ccc} \alpha_{11} & \ldots & \alpha_{d1}\\ \vdots & \ddots & \vdots \\ \alpha_{1d} & \vdots & \alpha_{dd} \end{array}\right]=\left[\begin{array}{ccc} x_{1}\left(1\right)-{\bar{x}}_{1+} & \vdots & x_{d}\left(1\right)-{\bar{x}}_{d+}\\ \vdots & \ddots & \vdots \\ x_{1}\left(N\right)-{\bar{x}}_{1+} & \vdots & x_{d}\left(N\right)-{\bar{x}}_{d+} \end{array}\right]. \end{array}$$

Denote by Λ the matrix $\left(\alpha_{ij}\right)$; then, the matrix of unknowns in the above equation sets is $\Lambda^{T}$. Left multiplication by

$$\begin{array}{c} {\left[\begin{array}{ccc} x_{1}\left(0\right)-{\bar{x}}_{1} & \ldots & x_{d}\left(0\right)-{\bar{x}}_{d}\\ \vdots & \ddots & \vdots \\ x_{1}\left(N-1\right)-{\bar{x}}_{1} & \ldots & x_{d}\left(N-1\right)-{\bar{x}}_{d} \end{array}\right]}^{T} \end{array}$$

on both sides yields *d* d×d equation sets:

(11) $$\begin{array}{c} C\Lambda^{T}=\tilde{C}, \end{array}$$

where $C=(C_{ij})$ is the sample covariance matrix of x, $\tilde{C}=\left(C_{i,j+}\right)$, and $C_{i,j+}$ is the sample covariance between $x_{i}$ and $x_{j+}$, i.e., $x_{j}$ advanced by one time step. The least-square solutions of the overdetermined sets (10) are the solutions of (11),

$$\begin{array}{c} \Lambda^{T}=C^{-1}\tilde{C}, \end{array}$$

and hence

$$\begin{array}{c} \Lambda ={\left(C^{-1}\tilde{C}\right)}^{T}={\tilde{C}}^{T}C^{-1}. \end{array}$$

The estimator of A is, therefore,

(12) $$\begin{array}{c} \hat{A}=\frac{1}{\Delta t}\log\left({\tilde{C}}^{T}C^{-1}\right). \end{array}$$

(Caution should be used in cases of singularity. The irrelevant imaginary part also should be discarded.)

Once we have obtained *A*, and hence the coefficients $\left(a_{ij}\right)$, we substitute $a_{ij}$ for the whole part

$$\begin{array}{c} \frac{1}{\det C}\sum_{k=1}^{d}\Delta_{jk}C_{k,di} \end{array}$$

in Equation (3), i.e., we multiply $a_{ij}$ by $C_{ij}/C_{ii}$ to arrive at the desideratum, ${\hat{T}}_{j\to i}$. If we denote by ${\left[A\right]}_{ij}$ the extraction of the ${\left(i,j\right)}^{th}$ entry of the matrix A, this is

(13) $$\begin{array}{c} {\hat{T}}_{j\to i}=\frac{1}{\Delta t}{\left[\log({\tilde{C}}^{T}C^{-1})\right]}_{ij}\cdot \frac{C_{ij}}{C_{ii}}. \end{array}$$

(Note here that log is the matrix logarithm. In MATLAB, the function is logm.)

## 4. The Coarsely Sampled Series Problem Revisited

As demonstrated above, for series generated from linear systems, the estimation of the IF is satisfactory qualitatively. Nevertheless, we want to examine how the new scheme may improve the results. Shown in Table 2 is a recalculation of the estimates. Since this case has a ground truth (half analytical) (≈0.11 nats per unit time), we can see that the result is accurate enough for all SIs.

**Figure 4:**
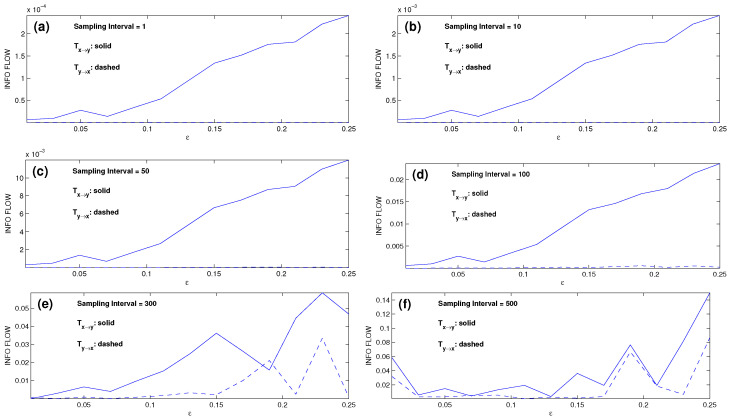
As in Figure 3, but the information flow rates are computed with the new scheme. Subfigures (**a**)–(**f**) correspond to the cases with SI = 1, 10, 50, 100, 300, 500, respectively.

The new scheme for the estimation is particularly satisfactory for the nonlinear case. For the pair of Rössler oscillators, the computed results are plotted in Figure 4. Compared to Figure 3, it can be seen that the performance is significantly improved. Consider the cases with SI ≤ 100 first. To see the improvement more clearly, we introduce a ratio $r=|T_{y\to x}\left|/\right|T_{x\to y}|$ to measure the performance of the one-way causal inference; the smaller the value, the more accurate the result, with r<0.1 taken as insignificant. By this standard, the cases with SI ≤ 10 (Figure 3a,b) appear satisfactory, but the causal relations as shown in Figure 3c,d are inaccurate or even incorrect. Specifically, for ε>0.15, i.e., when the system is synchronized, $r_{\max}$ reaches $\frac{1}{3}$ and $\frac{1}{2}$ for the cases of SI = 50 and SI = 100, respectively. For ε≤0.15, the inference appears satisfactory, albeit inaccurate. However, at ε=0.01, r≈1, the inference is incorrect. In contrast, the inferred causalities in Figure 4a–d are rather accurate for the coupling strengths ε considered (both synchronized and nonsynchronized), with all the *r*’s being insignificant.

For the case SI = 300, which corresponds to a sampling frequency of 20 points per period, the one-way causality is accurately recovered for the nonsynchronized cases (ε≤0.15). However, beyond this, i.e., ε>0.15, the inference fails. In particular, when SI = 500 (Figure 4f), the result is even worse than its counterpart obtained with the traditional scheme as plotted in Figure 3f. This, of course, may be due to the resulting small ensemble size, which causes singularity to the matrix logarithm. Consider the extreme limit when the series are completely synchronized. In this case, it is not possible to determine whether there exists a causal relation or not, as they are identical, impossible to be differentiated. Correspondingly, this means a singular covariance matrix. Now, in the above problem, the series are nearly synchronized. Given the length, the size of the ensemble thus formed reduces as the sampling interval increases. This tends to increase the condition number of the resulting covariance matrix. Whenever a matrix is ill-conditioned, its logarithm is very sensitive, leading to large errors in the subsequent computation.

Nonetheless, the success in applying the linear estimator to such a highly nonlinear system is remarkable. The reason for this is probably the same as that for linearization; that is to say, on a small interval, a linearized system can provide a good approximation to an otherwise nonlinear system. While its applicability is yet to be proven, or investigated with more nonlinear problems, by our experience, it indeed works well, provided that the statistics are sufficient (stochastic systems or chaotic deterministic systems) and the sampling interval is not too large.

## 5. Discussion

The maximum likelihood estimator of the information flow (IF), i.e., Equation (3), provides an easy way toward causal inference. Theoretically, it is based on a linear assumption, but, practically, it has shown considerable success with series generated from highly nonlinear systems; anyhow, linearization piecewise in time proves to be an efficient asymptote to an otherwise nonlinear system. In reality, series may be coarsely sampled; the time resolution may be low. An issue thus arises, as this formalism is theoretically on the basis of infinitesimal time increments. In this case, as we have shown, it still works for linear systems in a qualitative sense; however, for a highly nonlinear system composed of two Rössler oscillators, the bias becomes increasingly significant as the sampling frequency is reduced.

A new scheme has been proposed to address this problem and provide a partial solution. Due to a property of the IF, as proven in [7], that additive noise does not alter the IF in form, it is reasonable to directly estimate the IF without paying attention to the stochasticity. Instead of estimating through the vector field using Euler–Bernstein differencing, we choose to consider the propagator on the finite time interval, i.e., to estimate the Lie group members. In doing so, the original Formula (3), which is rewritten here for ease of reference,

$$\begin{array}{c} {\hat{T}}_{j\to i}=\frac{1}{\det C}\cdot \sum_{\nu =1}^{d}\Delta_{j\nu }C_{\nu ,di}\cdot \frac{C_{ij}}{C_{ii}}, \end{array}$$

is replaced with (13),

$$\begin{array}{c} {\hat{T}}_{j\to i}=\frac{1}{\Delta t}{\left[\log({\tilde{C}}^{T}C^{-1})\right]}_{ij}\cdot \frac{C_{ij}}{C_{ii}}, \end{array}$$

where $\tilde{C}=\left(C_{i,j+}\right)$, and $C_{i,j+}$ is the sample covariance between $x_{i}$ and $x_{j+}$, i.e., $x_{j}$ advanced by one time step. Note that here log is the matrix logarithm; in MATLAB (Version 23.2 or earlier), the function is logm. (The spurious imaginary part, if it arises, should be discarded.) In this way, the preset causality within the coupled system of chaotic oscillators can be rather accurately reproduced even when the sampling interval is large (sampling frequency is low). For convenience, this is summarized as an algorithm (Algorithm 1).
**Algorithm 1:** Causal inference through information flow estimation**input:** *d* time series and time step size Δt**output:** a causal graph G=(V,E) and IFs along edges    evaluate the covariance matrices C and $\tilde{C}$;    compute $P=\log({\tilde{C}}^{T}C^{-1})$;**for each** (i,j)∈V×V**do**        compute ${\hat{T}}_{i\to j}$ by (13), i.e.,        $\;{\hat{T}}_{j\to i}=\;\frac{1}{\Delta t}P_{ij}\;\cdot \frac{C_{ij}}{C_{ii}};$**end**return G, together with the IFs ${\hat{T}}_{j\to i}$

**Figure 5:**
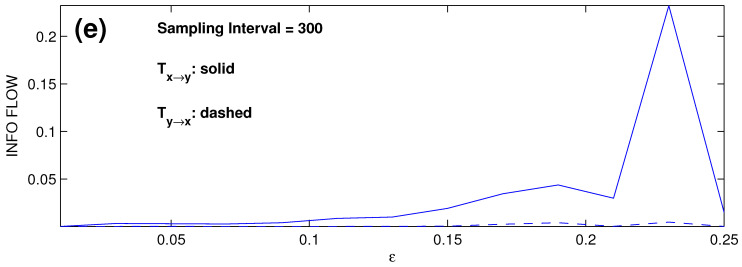
As in Figure 4e, but the covariances are estimated using the residuals of $x_{i}$ relative to the model result, instead of $x_{i}$ themselves. Here, SI = 300 implies a sampling frequency of 18 points each period. The label “(**e**)” in the figure correponds to the same lable in Figure 4e.

There is still much room for improvement in the above approach. For example, the estimation of the covariances in the quotient $\sigma_{ij}/\sigma_{ii}$ is achieved by replacing the population covariances with sample covariances, while the sample is formed from the time series. While this is satisfactory for stochastic systems under the ergodic assumption, this may not be suitable for deterministic chaos, such as in the case of the Rössler oscillators studied here. The reason is obvious: the time mean of the series in Figure 2 is zero, but one can imagine that the ensemble mean of all possible paths is by no means zero; rather, it should be a function of time (like the series itself), which may be close to the asymptotic linear system solution. Thus, it is more reasonable to treat the linear system solution as the mean. As such, we have attempted to improve the estimation by replacing the covariances of x with those of $x-\bar{x}$, where $\bar{x}$ stands for the resulting linear system solution. With this, we obtain another causal inference result for SI = 300; the resulting IFs are plotted in Figure 5. As one can see, the result appears rather accurate, as expected, in contrast to Figure 4e.

We, however, do not claim that we have solved the problem. What we want to show here is, how much room there is for improvement within a linear framework in estimating Equation (2), the original fully nonlinear formula $T_{j\to i}$. Indeed, in the case of high nonlinearity, the linear assumption is always easy to blame. While theoretically it is not a problem (causality is guaranteed as proven in a theorem; see [6] and other references), it is believed that, before a fully nonlinear algorithm is developed (when this paper was prepared, ref. [22] was not published), this will be a continuing issue.

It should be pointed out that the above algorithm works only when the sampling interval does not exceed the scale in question. For processes with multiple scales involved, different samplings may result in different physically meaningful information flow rates. In this case, it is not just a computational issue any more; it is a physical problem that deserves for an in-depth investigation. We will leave this problem, among others, for future research.

## Figures and Tables

**Table 1:** The estimated information flow rates within the linear system (4) for different sampling intervals (SIs). Also shown are the confidence intervals at a level of 90%.

S.I. (# of Points)	1	10	50	100	300	500
${\hat{T}}_{2\to 1}$	0.110 ± 0.051	0.113 ± 0.051	0.099 ± 0.050	0.090 ± 0.048	0.055 ± 0.036	0.055 ± 0.034
${\hat{T}}_{1\to 2}$	−0.002 ± 0.056	−0.001 ± 0.055	−0.015 ± 0.054	−0.011 ± 0.053	−0.008 ± 0.038	−0.015 ± 0.034

**Table 2:** Estimation of the information flow rates within the system (4) with the new scheme.

Sampling Interval	1	10	50	100	300	500
${\hat{T}}_{2\to 1}$	0.114	0.118	0.109	0.106	0.082	0.098
${\hat{T}}_{1\to 2}$	0.007	0.008	−0.007	−0.002	−0.002	−0.015

## Data Availability

The codes are available, and will be updated, at www.ncoads.org/article/show/67.aspx (accessed on 1 January 2020).
